# From the principles of genomic data sharing to the practices of data access committees

**DOI:** 10.15252/emmm.201405002

**Published:** 2015-03-10

**Authors:** Mahsa Shabani, Bartha Maria Knoppers, Pascal Borry

**Affiliations:** 1 Centre for Biomedical Ethics and Law, Department of Public Health and Primary Care, University of LeuvenLeuven, Belgium; 2 Centre of Genomics and Policy, McGill UniversityMontreal, QC, Canada

## Abstract

Sharing genomic research data through controlled-access databases has increased in recent years. Policymakers and funding organizations endorse genomic data sharing in order to optimize the use of public funds and to increase the statistical power of databases. Well-established data access arrangements and data access committees (DACs)—responsible for reviewing and managing requests for access to genomic databases—are therefore central for implementing the policies and principles of data sharing. This article aims to investigate the functionality of DACs through the perspective of existing practices.

Data access committees (DACs) are an integral component of managing access to genomic databases. DACs are responsible for reviewing, approving or disapproving requests from potential users for a variety of controlled-access genomic databases (Fortin *et al*, 2010; Kaye & Hawkins, 2014). Owing to the complexity of studies and various ethical and legal issues, DACs usually are independent commissions, rather than individual researchers who decide who gets access: “the tradition in which custodian principal investigators themselves made access decisions has generally been giving way to more consultative decision-making with independent input” (Lowrance, 2006). In fact, an independent DAC is better placed to ensure fair and informed decision-making about data access. This includes addressing concerns regarding the potential force identifiability of genomic data, adequacy of the original consent for collected data, the quality of the data and protecting data producers' publication rights while enabling a timely and broad access to databases for many users (Kaye *et al*, 2009; O'Brien, 2009; McGuire *et al*, 2011).

DACs, however, function in different ways. While some large research or funding organizations such as the US National Institute of Health (NIH) constructed their own databases and use central DACs to manage access requests, other DACs are located within study groups or consortia. An overview of the current practices of DACs reveals that some aspects need to be improved in order to benefit the ultimate goal of sharing genomic research data.

## Heterogeneous or underdeveloped access arrangements

Access to data sets in some public databases, such as the European Genome-phenome Archive (EGA), is managed through local DACs in a decentralized fashion. For users, this means that they have to adhere to a variety of access arrangements adopted by each DAC. In the absence of established guidelines and criteria, one can expect a considerable plurality in the practices of DACs. A preliminary review of 212 DACs listed in the EGA reveals that only a few of these committees are sufficiently described on the corresponding EGA web page. In the majority of cases, it provides only the information for a DAC's contact person, which makes it difficult if not impossible to investigate the membership of the DAC, or its guidelines and the procedures it uses to evaluate access requests. This begs the question whether such scarcity of information is an indication of underdeveloped access arrangements or a lack of proper communication of existing arrangements. In any sense, this lack of transparency may fail to adequately inform data users how DACs assess requests, thus adversely affecting data sharing practices. In comparison with single or small studies, DACs for institutions and genomic consortia make general information more readily available on their web pages or through the relevant publications.

Lack of funding and motivation is probably the reason for the limited investment in developing and communicating adequate access arrangements for small studies. Funding agencies could therefore take the lead in designing adequate data access arrangements or developing central DACs with adequate financial support to comply with those arrangements. The database of genotypes and phenotypes (dbGaP), designed to store results of NIH-sponsored human genomic studies, exemplifies this centralized approach. Within this database, 16 DACs “review requests for consistency with any data use limitations and approve, disapprove or return requests for revision”, except for large studies in which a local DAC leads the review (Paltoo *et al*, 2014). Small studies could thus benefit from a central DAC infrastructure that follows the central access arrangements policies, such as the recent NIH policy for genomic data sharing (http://gds.nih.gov/PDF/NIH_GDS_Policy.pdf). Collaboration of small DACs to “develop regional or national policy consortia” would be an alternative solution (Lemke *et al*, 2011). Nevertheless, the interests and concerns of researchers differ from one study to another and must be considered when adopting a centralized approach to data access. Moreover, a complex or cumbersome access arrangement that is not designed properly could also impede the efficiency of a centralized model.

Harmonization of data sharing practices has emerged as a priority in data-intensive research on an international level. Biobanking and Biomolecular Resources Research Infrastructures and the international Public Population Project in Genomics and Society (P3G) are international initiatives that offer tools to facilitate governance of genomic data sharing and global access to databases. P3G has prepared a model of generic access agreements to address issues relevant to delivery, privacy, security, liability, intellectual property, publication, reporting and termination of the agreement (Knoppers *et al*, 2013). In the same line, the Global Alliance for Genomics and Health has adopted a *Framework for Responsible Sharing of Genomic and Health-Related Data* to support an international agenda for data sharing (Knoppers, 2014). Although the harmonization of data access agreements would facilitate data access at a global level, it should consider local legislation with respect to processing personal data and the responsibilities of data custodians, which may vary across jurisdictions.

Finally, procedures to evaluate qualified researchers and acceptable research studies are not often well-defined in data access arrangements. Data users may be asked to provide contact information, a list of recent publications and a description of the proposed research. Delineation of such criteria by DACs is important to ensure the fairness of access assessment procedures. Concurrently, the independence of the DACs should promote neutral evaluation of data requests separate from personal considerations between data producers and users, especially considering potential conflict of interests. This highlights a need for an entity to which complaints about DAC decisions can be referred to.

Beyond the data producers and the users' interests and concerns, the pertinent data access arrangements should also be attentive to the interests and concerns of data subjects. To this end, it is pivotal to obtain more insights about the concerns of data subjects on an individual or a group level in order to inform the future data access decisions by DACs. The significance of such inquiries is accentuated given the shortcomings of traditional mechanisms such as one-time broad consent where group-based considerations are rarely addressed.

## Lack of sufficient oversight mechanisms

In principle, duties and responsibilities of data users are articulated through contractual agreements, where failure to comply sets legitimate grounds to revoke access permissions (Joly *et al*, 2011). These agreements encompass various aspects of data use, ranging from security of data storage to the publication of results, in order to respect the rights of research participants and data producers. Moreover, DACs can require compliance with certain standards to assure that effective security protection measures are in place including physical protections, administrative discipline and cyber security (Lowrance, 2012).

Currently, oversight mechanisms over enforcement of agreements and standards are not thoroughly elaborated. Some data access policies already consider issues such as auditing data use by DACs and handling reports from users. Yet, such sporadic approaches fail to meet needs for consistent and ongoing oversight. Owing to the distance between data producers and data users—and the potential lack of expertise within DACs to deal with technical features of data security systems in various institutions—the feasibility of an effective and robust mechanism for data access oversight is questionable (Kaye *et al*, 2009). Various regulatory measures in different countries regarding personal data protection and secondary use of research data also make it harder to perform efficient oversight on data use across countries.

To date, a few cases of violation of the contractual agreements have been reported, which mainly resulted from disrespecting publication policies such as publication embargoes (Holden, 2009). Subsequently, this highlights the role of journal editors or reviewers to identify infringements in using genomic research data (Nanda & Kowalczuk, 2014). The complexity of data use arrangements makes it a shared responsibility of all relevant parties to ensure that publication requirements are communicated clearly and followed up. In addition, it would be helpful to analyse whether data sharing practices have so far generated any other material or moral harm to the involved parties. Not least for the sake of such investigation, identifying violators of access arrangements and notifying stakeholders of breaches should be streamlined. The results of such investigation will assist to fine-tune the current oversight mechanisms and sanctions if needed. In the absence of such ongoing overview, it is possible that the current protective mechanisms are not adequate to deal with the magnitude and scope of the associated risks.

## Lack of clarity in relationships between DACs and ethics committees

The relationship between DACs and institutional review boards/research ethics committees (IRBs/RECs) should be better defined and more transparent. DACs often refrain from adding another layer of ethics review, seeing it as the responsibility of data users to satisfy the requirements for ethics approval. In some instances, DACs require an official ethics approval document from the home institution. On some occasions, DACs have undertaken further evaluation of the proposed research uses, particularly when there are ethical concerns. It is not clear, however, whether these DACs intend to systematically develop an ethical review or refer specific cases to the ethics committees at researchers' home institutions, or request further evaluation by the data user's institution. In any event, DAC's review should dovetail with that of IRBs/RECs to avoid redundancies. Again, guiding principles and standards in order to streamline the practices and involvement of ethics committees at an international level could help to better define this relationship.

Finally, growing data-intensive research illustrates the need for and value of globally accessible data. This will transform current approaches to review and the responsibilities of IRBs/RECs and DACs. An optimal review should be responsive and proportionate to the particularities of research with genomic data, which differ from the physical risks associated with, say, clinical trial studies. Designing access reviews regardless of the type of data and the risks and benefits associated with the research proposal contradicts the principle of proportionality. It is therefore crucial to develop clear criteria of risks associated with genomic research. This tailored approach to review depends on the sensitivity of the data used and mechanisms to safeguard privacy and confidentiality.

Developing thorough and efficient data access arrangements is a key to promoting research that uses genomic data. DACs have a critical role in implementing arrangements that are framed in accord with the overarching principles of genomic data sharing. In order to approve access requests to controlled-access databases, DACs should aim for consistency. Transparent guidelines and criteria for qualified researchers and research purposes should be set and communicated adequately. Guidelines and policy statements are well placed to promote best practices, particularly where local regulations do not address the associated issues or there is a need for clarifications. In essence, general guidelines are valued as complementary documents prepared for advisory purposes. Benefiting from professional and expert groups expertise, guidelines could provide practical recommendations in leading data submission, storage and distribution. Involving various stakeholders and seeking a broader consensus will ensure the comprehensiveness of the guidelines in tackling data sharing concerns. It would also require oversight mechanisms to ensure that data users and producers adhere to such guidelines.

**Suggested Minimal Guidelines**

- Developing access arrangements and communicating them sufficiently to data users will reinforce transparency and facilitate wider access to databases.
- A deeper involvement of funding organizations in developing access arrangements and setting up data access committees would be especially beneficial for small studies with limited resources.
- Harmonization of data access arrangements is necessary for successful international data sharing and to ensure fairness of the procedure.
- Data subjects' concerns at the individual and group levels should be identified and respected.
- Oversight mechanisms on the enforcement of data access agreements and standards should be elaborated and arrangements made for detection and sanction of violations.
- To avoid redundancies, the relationship between data access committees and other oversight bodies such as ethics committees and the scope of their oversight should be clarified.
